# Automatic Figure Ranking and User Interfacing for Intelligent Figure Search

**DOI:** 10.1371/journal.pone.0012983

**Published:** 2010-10-07

**Authors:** Hong Yu, Feifan Liu, Balaji Polepalli Ramesh

**Affiliations:** 1 Department of Health Sciences, University of Wisconsin-Milwaukee, Milwaukee, Wisconsin, United States of America; 2 Department of Electrical Engineering and Computer Science, University of Wisconsin-Milwaukee, Milwaukee, Wisconsin, United States of America; 3 Medical Informatics, University of Wisconsin-Milwaukee, Milwaukee, Wisconsin, United States of America; RMIT University, Australia

## Abstract

**Background:**

Figures are important experimental results that are typically reported in full-text bioscience articles. Bioscience researchers need to access figures to validate research facts and to formulate or to test novel research hypotheses. On the other hand, the sheer volume of bioscience literature has made it difficult to access figures. Therefore, we are developing an intelligent figure search engine (http://figuresearch.askhermes.org). Existing research in figure search treats each figure equally, but we introduce a novel concept of “figure ranking”: figures appearing in a full-text biomedical article can be ranked by their contribution to the knowledge discovery.

**Methodology/Findings:**

We empirically validated the hypothesis of figure ranking with over 100 bioscience researchers, and then developed unsupervised natural language processing (NLP) approaches to automatically rank figures. Evaluating on a collection of 202 full-text articles in which authors have ranked the figures based on importance, our best system achieved a weighted error rate of 0.2, which is significantly better than several other baseline systems we explored. We further explored a user interfacing application in which we built novel user interfaces (UIs) incorporating figure ranking, allowing bioscience researchers to efficiently access important figures. Our evaluation results show that 92% of the bioscience researchers prefer as the top two choices the user interfaces in which the most important figures are enlarged. With our automatic figure ranking NLP system, bioscience researchers preferred the UIs in which the most important figures were predicted by our NLP system than the UIs in which the most important figures were randomly assigned. In addition, our results show that there was no statistical difference in bioscience researchers' preference in the UIs generated by automatic figure ranking and UIs by human ranking annotation.

**Conclusion/Significance:**

The evaluation results conclude that automatic figure ranking and user interfacing as we reported in this study can be fully implemented in online publishing. The novel user interface integrated with the automatic figure ranking system provides a more efficient and robust way to access scientific information in the biomedical domain, which will further enhance our existing figure search engine to better facilitate accessing figures of interest for bioscientists.

## Introduction

Research in bioscience figures has gained much attention recently [1]–[10]. Figures are usually the “evidence” of bioscience experiments [1]. Researchers need access to figures to validate research facts and to formulate and test novel research hypotheses. In addition, with more and more genome-wide data being made publicly available (e.g., Gene Expression Omnibus) and ever-increasing numbers of computational approaches for predicting findings and hypotheses, examining figures reported in bioscience literature remains one of the most effective approaches for validating the predictions.

On the other hand, mining knowledge from bioscience figures is a very challenging task. First, the semantics of bioscience figures are extremely rich and require the mining of both the image features themselves [2] and the associated text [1], [7], [8], [11], [12]. Secondly, bioscience figures are abundant (we found an average of over 5 figures per bioscience article in *Proceedings of the National Academy of Sciences* [1]). Furthermore, figures within the same article are not isolated, but are semantically related [1]. To discover and utilize the relationships among figures is quite crucial for knowledge mining from bioscience figures.

However, nearly all research to date in knowledge mining from bioscience figures, such as figure search engines [7], [8] embraces a “bag of figures” assumption, which leads to the loss of useful information. In this paper we depart from such a semantically lean approach to explore the relationships among bioscience figures and novel user interfaces of information access.

We describe a novel hypothesis for semantically relating figures in bioscience literature: that those figures can be ranked in terms of their bio-importance. We empirically validate this hypothesis and propose natural language processing (NLP) approaches for the automation of figure ranking. We also developed and evaluated novel user interfaces that are built upon figure ranking. Our work is developed in the context of building an intelligent bioscience figure search engine.

## Materials and Methods

### Background and A Novel Hypothesis

Our previous work has shown that figures appearing in a full-text bioscience article semantically associate with sentences in the abstract [1]. We examined different types of associated text (title, abstract, figure legends, and associated text appearing in the body) [11] and evaluated their contribution to figure comprehension [12]. We also developed summarization methods for aggregating distributed associated text, removing redundant text, and automatically generating a structured text summary for every figure [13], [14]. Our fully implemented figure search system (http://figuresearch.askhermes.org) has been evaluated and used by over one hundred bioscience researchers.

In this work, we argue that figures appearing in bioscience articles differ in their importance. While some figures may play a supportive role (e.g., steps of an experimental protocol), others may represent key knowledge discoveries. We hypothesize that figures appearing in a bioscience article can be ranked by their importance.

### Hypothesis Testing for Figure Ranking and Gold Standard

To test whether bioscience figures can be ranked, we asked biologist authors to rank figures in their publications. We randomly selected 1,750 bioscience articles which were most recently published (year 2003–2009) in four journals (*Cell* (199), *Journal of Biological Chemistry* (371), *Proceedings of the National Academy of Sciences* (197), and *PLoS Biology* (983). Those articles were published by authors in 39 countries, with the highest numbers (656 and 153) published in United States and United Kingdom, and the lowest number (1 article), which was published by authors in 13 countries, including Chile, Denmark, and Hungary. For each article we emailed the corresponding author, asking them to rank figures in their publication by their biological importance.

**Figure 1:**
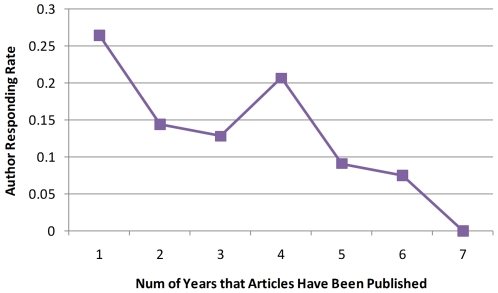
Author's responding rate as a function of number of years that articles have been published. For example, “1” indicates that articles were published in 2009 and “7” indicates that articles were published in 2003.

Until this study, a total of 298 authors from 22 countries responded to our email requests. The responding rate ranged from 5% (authors in France) to 100% (authors in Chile, Denmark, and Hungary). The responding rates for authors in UK and USA were 13.7% and 10.8%. We speculate that there is a reverse relation between author responding rate and the “age” of publication, and therefore plot Figure 1, which shows author responding rate by the number of years that articles have been published. Of the total 298 responded authors, a minority of them (46 or 15.4%) stated that either figures could not be ranked, or the ranking depended upon specific research interests. In contrast, a majority of authors (252 or 84.6%) ranked the figures in their publications.

The 252 annotated articles were used for evaluating new user interfaces reported in this study (described in more detail in user interface subsections below). However, of the 252 articles, we could download automatically only 202 full-text articles. This collection of 202 annotated full-text articles was used to evaluate our NLP approaches for automatic figure ranking as described in the following section. For those 202 articles, the average number of figures was 5.9
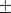
1.75 (range: 2 in [15] to 13 in [16]).

### NLP Approaches for Figure Ranking

We explored natural language processing (NLP) approaches for automatic figure ranking. We hypothesize that the most important figure should be the focus or the central point of the full-text bioscience article. The ranking can thus be determined by its degree of centrality in the context of summarization [17], which indicates how closely this figure represents the main findings of the article. In our study, we assume that a figure's content is represented by its associated text, which includes figure caption and other associated text in which the figure is mentioned. Such associated-text representation of figure content has been evaluated and validated in a number of studies [1], [11], [12].

### System Description

We modeled the degree of centrality of each figure by calculating the similarity between a figure and the full-text article in which the figure appears. Two figure representations were explored, one in which a figure was represented by the text in its legend, *FIGlegend*, and another where it is represented its associated text in the article, *FIGtext*. The degree of centrality of each figure can be thought as a lexical distance between the *FIGlegend* or *FIGtext* and the article summary, for which three representations were explored, corresponding title (*ATCtitle*), abstract (*ATCabstract*), and full text (*ATCtext*). The lexical similarity is calculated with the vector-space model [18], an information retrieval model which calculates the TF*IDF-weighted cosine similarities were calculated over the 19 million MEDLINE records.

We took as input the original HTML format of each article. We used the Lynx tool (http://lynx.isc.org) to strip the text information and performed the tokenization with TreeTagger (http://www.ims.uni-stuttgart.de/projekte/corplex/TreeTagger). We then extracted *FIGlegend*, *FIGtext*, *ATCtitle*, *ATCabstract* and *ATCtext* with hand-coded regular expression. We determined the ranks of figures based on the degree of centrality, with higher rank assigned to the figure with the larger cosine similarity score described above. Note that different representations of figures and articles would lead to six different ranking systems, as shown in Table 1.

**Table 1:** Performance of automatic figure ranking based on similarities between different representation of figures and articles.

System	MER	MWER	MWER-RK	ER-HR	WER-HR
*FIGlegend-ATCabstract*	0.416	0.366	0.265	0.649	0.283
*FIGlegend-ATCtitle*	0.442	0.399	0.283	0.660	0.287
*FIGlegend-ATCtext*	0.436	0.385	0.280	0.671†	0.304†
*FIGtext-ATCabstract*	**0.378** ‡	**0.322** ‡	**0.223** ‡	0.616§	0.266‡
*FIGtext-ATCtitle*	0.399	0.362	0.255	**0.594**	**0.246**
*FIGtext-ATCtext*	0.377†	0.321†	0.232	0.627	0.274

Significance level of T test compared to the first row is noted by

† (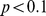),

‡ (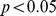) and

§ (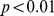) respectively.

We also examined frequency-based approach to measure the degree of centrality of each figure, where figures that are more frequently referred are considered as more important. In this framework, we evaluated ranking figures based on the following six strategies:

- *FreqFullText*
  Frequency in the full article. Figures are ranked simply based on the number of times they are referred to in the full text.
- *FreqRD*
  Frequency in the results and discussion sections. We hypothesize that the results and discussion sections contain relatively important information for figure comprehension. We applied regular expressions to deal with the name variations of these two sections in different journals.
- *WFreqRDParaTitle*
  We identify in results and discussion sections the most topic-relevant paragraph that refers to each figure and rank figures based on the frequency in this paragraph weighted by multiplying the relevancy score. We determine the relevancy using the cosine similarity between each paragraph and the article title, as described above, which is assigned to the corresponding frequency as a weight.
- *WFreqRDParaAbs*
  Unlike *WFreqRDParaTitle*, this method assigns the relevancy using the cosine similarity between each paragraph and the article abstract.
- *WFreqRDTitle*
  Figures are ranked by interpolating frequency in all the results and discussion paragraphs, weighted by the relevancy score of each paragraph with respect to the article title.
- *WFreqRDAbs*
  Unlike *WFreqRDTitle*, this method assigns frequency in different paragraphs by their relevancy score with respect to the article abstract.

### Evaluation Metrics

Figure ranking presents a new and unique NLP task and we explored different evaluation metrics. We first adopted the mean error rate (MER) and the mean-weighted error rate (MWER) [19], for the evaluation. For this specific task, MER measures the percentage of figure pair relations that are wrongly recognized and MWER assigns the weight to each wrongly recognized figure pair based on their distance in the reference ranking order. These metrics are defined as:
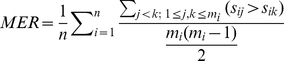
(1)

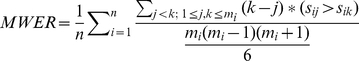
(2)where 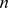 is the number of articles, 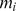 is the number of the figures in the 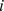
th article, 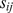 and 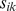 are the system ranks of figure 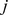 and 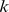 respectively in the 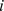
th article, 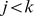 indicates reference ranking relationship between figure 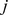 and 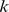.

However, MER and MWER do not take the figure's absolute ranking position into consideration. For example, suppose that the gold standard of a figure ranking in an article is (1,2,3,4). Given two system outputs (2,1,3,4) and (1,2,4,3), both MER and MWER assign the same error rates to the two outputs (0.167 and 0.1, respectively). However, a wrongly ranked figure pair should be given more penalties if the figure has a higher reference rank. In this example, the error rate for the output of (2,1,3,4) should be higher than the error rate of (1,2,4,3). This is especially evidenced in our novel user interface design section in which we need to judge the most important figures. We therefore define a new metric, MWER-RK, that considers the rank information. MWER-RK adds a logistic function to MWER that allows the metric to take into account the reference rank information involved in wrongly recognized figure pairs as shown below.
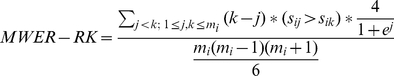
(3)


The higher the rank information is involved, the higher the weight the pair will get. With MWER-RK, the evaluation scores of (2,1,3,4) and (1,2,4,3) in the above example are 0.108 and 0.019, respectively.

We used the error rate of the highest rank(ER-HR) to evaluate identifying the most important figure:
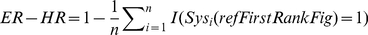
(4)where 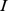
() is the indicative function with the value of 1 when the system output agrees with the reference on the highest rank figure and the value of 0 otherwise, *Sys* 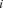
(*refFirstRankFig*) is the system output rank of the most important figure. Similarly, we defined a weighted error rate (WER-HR) for identifying the most important figure, which takes into account the distance between the annotated reference rank of the most important figure (rank 1) and the system's rank of it, i.e. the longer distance it is the more weight it would get, as shown below.
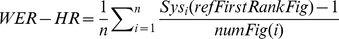
(5)where 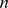 is the number of articles, *numFig*(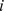) is the number of figures in the 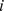
th article.

## Results

### Figure Ranking Results

Tables 1 and 2 report the figure ranking results with the similarity and frequency-based approaches, respectively. The results show that the best MWER-RK of 0.223 was achieved when using the similarity between associated text of the figure, *FIGtext*, and the article abstract, *ATCabstract*. In general, we found that the performance using the figure text – *FIGtext* (the last three rows in Table 1) was superior to the performance using the legend text – *FIGlegend* (the first three rows). In addition, representing an article by its abstract can produce better performance than by the title and full text. In terms of identifying the most important figures, “*FIGtext–ATCtitle*” obtained the best performance of 0.594 (ER-HR) and 0.246 (WER-HR).

**Table 2:** Performance of automatic figure ranking using frequency-based centrality.

System	MER	MWER	MWER-RK	ER-HR	WER-HR
*FreqFullText*	0.387	0.331	0.249	0.682	0.277
*FreqRD*	0.39	0.337	0.252	0.682	0.280
*WFreqRDParaTitle*	0.417†	0.369†	0.264	0.671	0.251
*WFreqRDParaAbs*	0.425‡	0.365‡	0.262†	0.693	0.298
*WFreqRDTitle*	0.389	0.340	0.245	**0.638**	**0.246** †
*WFreqRDAbs*	**0.379**	**0.319**	**0.228** †	0.649	0.249‡

Significance level of T test compared to the first row is noted by

† (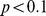),

‡ (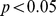) and

§ (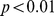) respectively.

The results show that the weighted frequency-based approach achieved MWER-RK of up to 0.228 (last row of Table 2) when weighted by the relevancy score with respect to the article's abstract. This approach also yielded the best performance for both MER(0.379) and MWER(0.319). We noticed that the system based on full text frequency(*FreqFullText*) resulted in the MWER-RK of 0.249 and WER-HR of 0.277, one of the top performances. Limiting the frequency information to result and discussion(R&D) sections(*FreqRD*) only did not help the performance. In contrast, when further taking into account most topic relevant paragraphs(*WFreqRDParaTitle* and *WFreqRDParaAbs*), relevancy analysis based on title is shown to be helpful(*WFreqRDParaTitle*) for recognizing the most important figures, with a 9.4% improvement on the WER-HR from 0.277 to 0.251. Interpolating frequency in R&D paragraphs(*WFreqRDTitle* and *WFreqRDAbs*) based on different weights, which were measured by topic similarity with the title or abstract, significantly outperformed their non-interpolated counterparts, leading to the best MWER-RK of 0.228 from *WFreqRDAbs* versus 0.262 from *WFreqRDParaAbs* and the best WER-HR of 0.246 from *WFreqRDTitle* versus 0.251 from *WFreqRDParaTitle*. Similar to Table 1, *WFreqRDTitle* performed better than *WFreqRDAbs* on recognizing the most important figures, yielding the ER-HR of 0.638 vs. 0.649 and the WER-HR of 0.246 vs. 0.249 (last two rows of Table 2).

**Figure 2:**
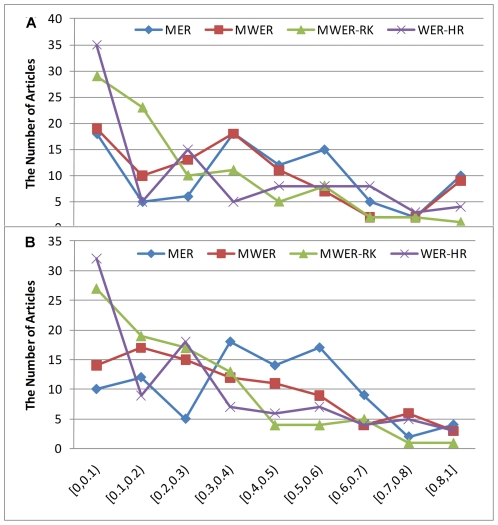
Number of articles as a function of the error rate based on different metrics. “A” for the best similarity based system *FIGtext*–*ATCabstract* and “B” for the best frequency based system *WFreqRDAbs*.

Figure 2 shows the distribution of number of articles as a function of weighted error rates for the two best systems based on similarity(*FIGtext*–*ATCabstract*) and frequency(*WFreqRDAbs*) respectively. Both figures show that a good portion of articles are predicted perfectly in figure ranking by our systems. The perfectly predicted articles include [20] which incorporates 7 figures with the order of importance {6,7,3,1,2,4,5}. Overall, the number of articles decreases when the error rate for each article increases. For the similarity-based approach (Figure 2A), the proportion of articles based on MWER is larger than on MER at lower error rates(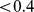) and it turns opposite at higher error rates(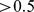). MWER-RK metric shows even better distribution with a larger number of articles than both MWER and MER at lower error rate(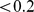) and decreasing number of articles as error rates increase(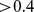) compared to those other two(MWER and MER). Similar trends are observed in the frequency-based approach (Figure 2B), but we do find different patterns than in the similarity-based approach. For example, the number of articles that had the highest MER or MWER [0.8,1] was cut in half, the number of articles with MWER less than 0.3 was increased, the number of articles with MWER-RK less than 0.5 was increased, and the number of articles with WER-HR larger than 0.5 was decreased. Those observations motivated us to build an integrated system, where the measurements from two systems (*FIGtext*–*ATCabstract* and *WFreqRDAbs*) are linearly combined for the final ranking decision, as follows:
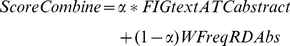
(6)where 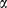 is the adjustable parameter to balance the contribution of two systems.

**Figure 3:**
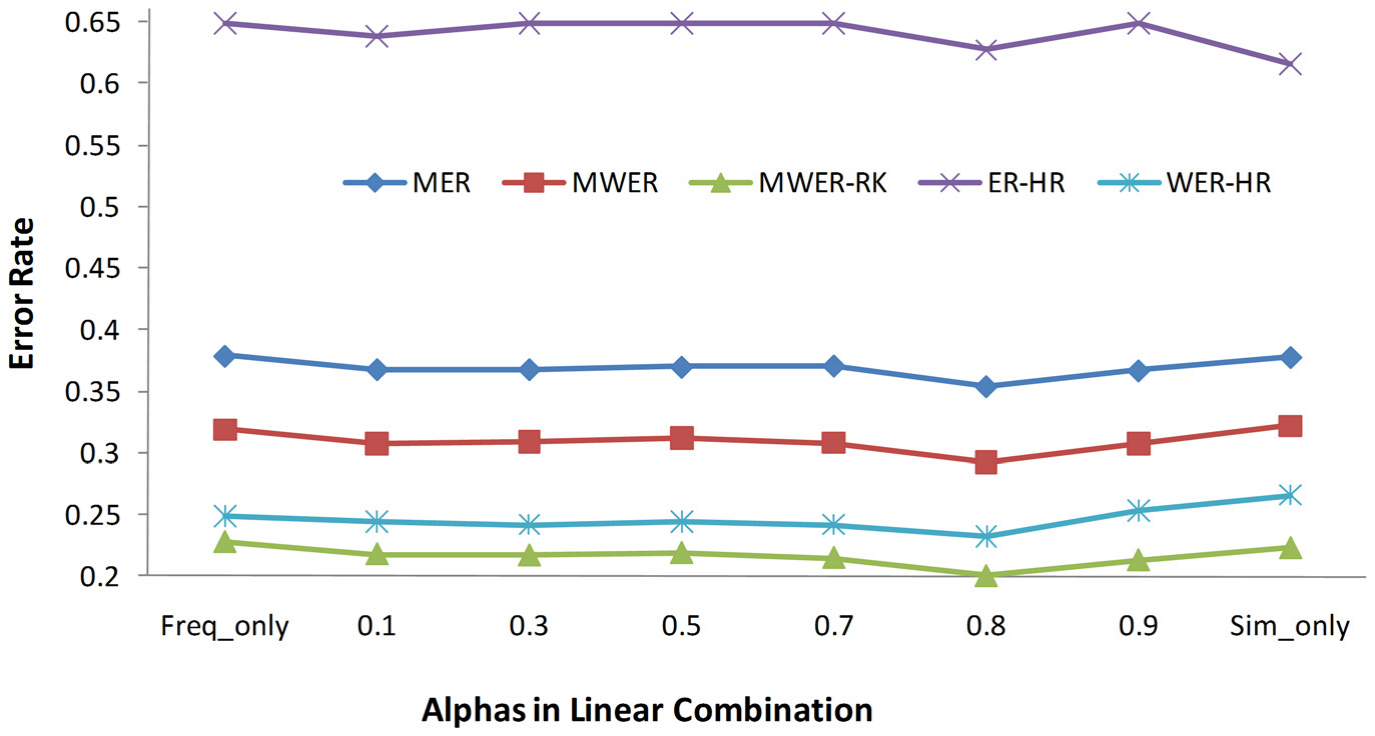
Performance curve with different 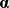
's in the linear combination. 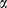
 being 0 corresponds to frequency based system and 1 corresponds to the similarity based system.

As shown in Figure 3, linear combination can further improve the overall performance by yielding the best error rates of 0.354 (MER), 0.292 (MWER) and 0.2 (MWER-RK) when 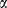 = 0.8, much better than using *FIGtext*–*ATCabstract* (0.378, 0.322 [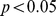], and 0.223 [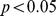], respectively) or *WFreqRDAbs* alone(0.379, 0.319, and 0.228, respectively). The combined system didn't show improved performance on ER-HR(0.627) compared to 0.616 of *FIGtext*–*ATCabstract*(not statistically significant with p value of 0.41), but it did show much better performance on WER-HR, yielding the value of 0.232 compared to 0.249 of *WFreqRDAbs* (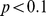) and 0.266 of *FIGtext*–*ATCabstract*(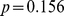). This also outperformed the best WER-HR of 0.246 we got previously in (Table 1 and 2), although not on ER-HR at which *FIGtext*–*ATi* system(Table 1) performed best at 0.594(not statistically significant).

We also conducted a pairwise two-sample two-tailed t-test on the average figure ranking performance by their Journal types (JBC, PLoS, PNAS). Our results show that there is no significant difference(p values range from 0.833 to 0.748) in terms of 5 metrics shown in Table 1 and 2. To obtain a further understanding of our experimental results, we performed error analysis, as shown in the following section.

### Error Analysis

There are different approaches for error analyses. In this study, we mainly focused on analyzing the wrong predictions of the most important figure, as they would play a pivotal role for our user interface, to be described in the next section. We observed that for many articles it may be a challenging task for figure ranking. Examples are shown below.

#### (1) Model

**Figure 4:**
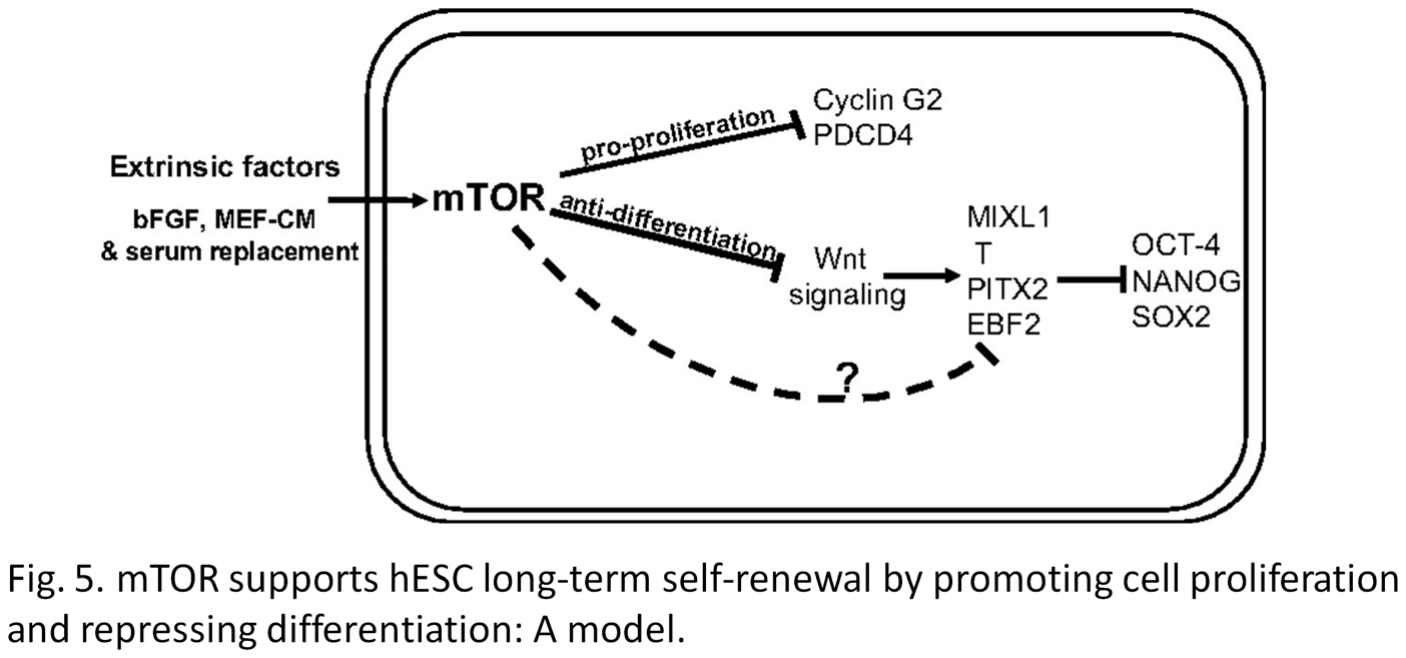
A model that appears in the article [**21**] as “**Fig. 5**”. The author did not judge this model as the most important figure, while other authors judged models in their publications as the most important figure.

Model figures (e.g., Fig. 5 of [21] as shown in Figure 4) are frequently introduced by bioscience researchers who summarize the discoveries in their articles and make new hypothesis. Some authors (e.g., [21]) judged the experimental evidence to be more important than models, while others (e.g., [22]) considered the final diagrams or models as the most important figures. Such inconsistency in annotation leads to decreased performance of our automatic figure ranking systems.

**Figure 5:**
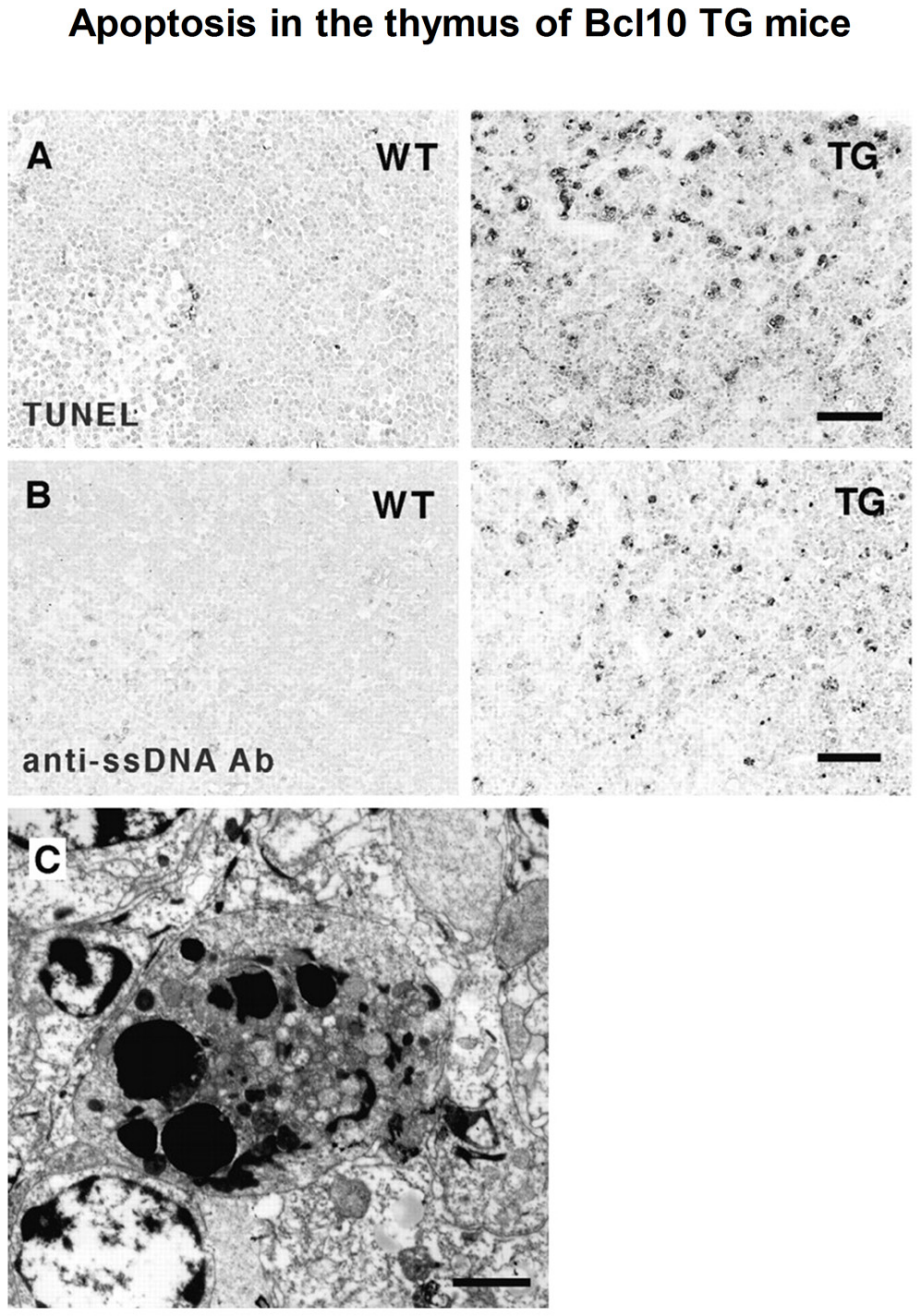
Different results leading to the same conclusion in one figure. In this single figure from article [24], results from three different assays, “TUNEL” “anti-ssDNA” and “electron microscopy” are depicted.

#### (2) Which is more important: concept generation or knowledge discovery?

Some authors think that the initial experiment that leads to hypothesis generation is most important, while others consider the core experiments or figures that lead to the main conclusion of the article to be most important. For example, the authors of [23] considered the most important figure in their paper to be “Fig. 1”, which shows that A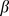 peptides significantly inhibit neurite outgrowth in p75 mutant sympathetic neurons, and the results suggest that p75 plays a role in attenuating A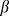-mediated inhibition of nerve growth. Our system predicted “Fig. 4” as the most important figure because it shows p75 reduces 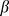-amyloid-induced sympathetic innervation deficits in an Alzheimer's disease mouse model, which we agree with our system on considering it to be the central point of the article.

#### (3) How to rank the importance of two complimentary experiments?

**Figure 6:**
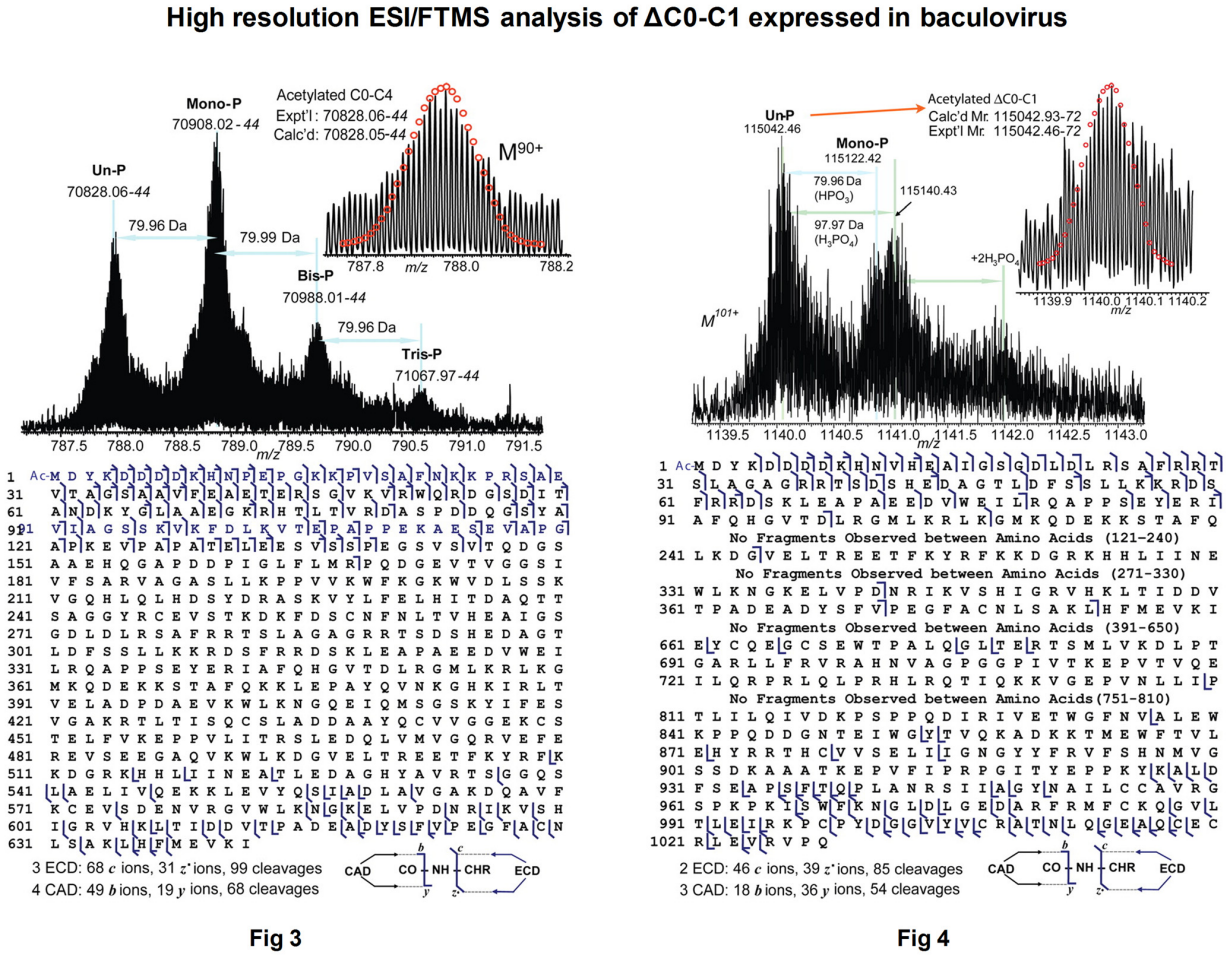
Two complimentary experiments in separate figures. “Fig. 3” and “Fig. 4” appearing in the article [25].

Bioscience discovery frequently involves multiple experiments. While in some cases experiments that lead to the same conclusion are presented in one figure (Figure 5 shows such an example in article [24]), in other cases experiment results are presented in multiple figures. For example, Figure 6 shows two figures, “Fig. 3” and “Fig. 4”, that appear in the article [25]. Both figures apply “high-resolution ESI/FTMS analysis”. “Fig. 3” analyzes C0-C4 expressed in baculovirus, while “Fig. 4” analyzes the deletion of C0-C1 in baculovirus, and the results support each other. In this example, the author of the article judged that “Fig. 4” is the most important, while our system ranked “Fig. 3” as most important.

### User Interface Evaluation

**Figure 7:**
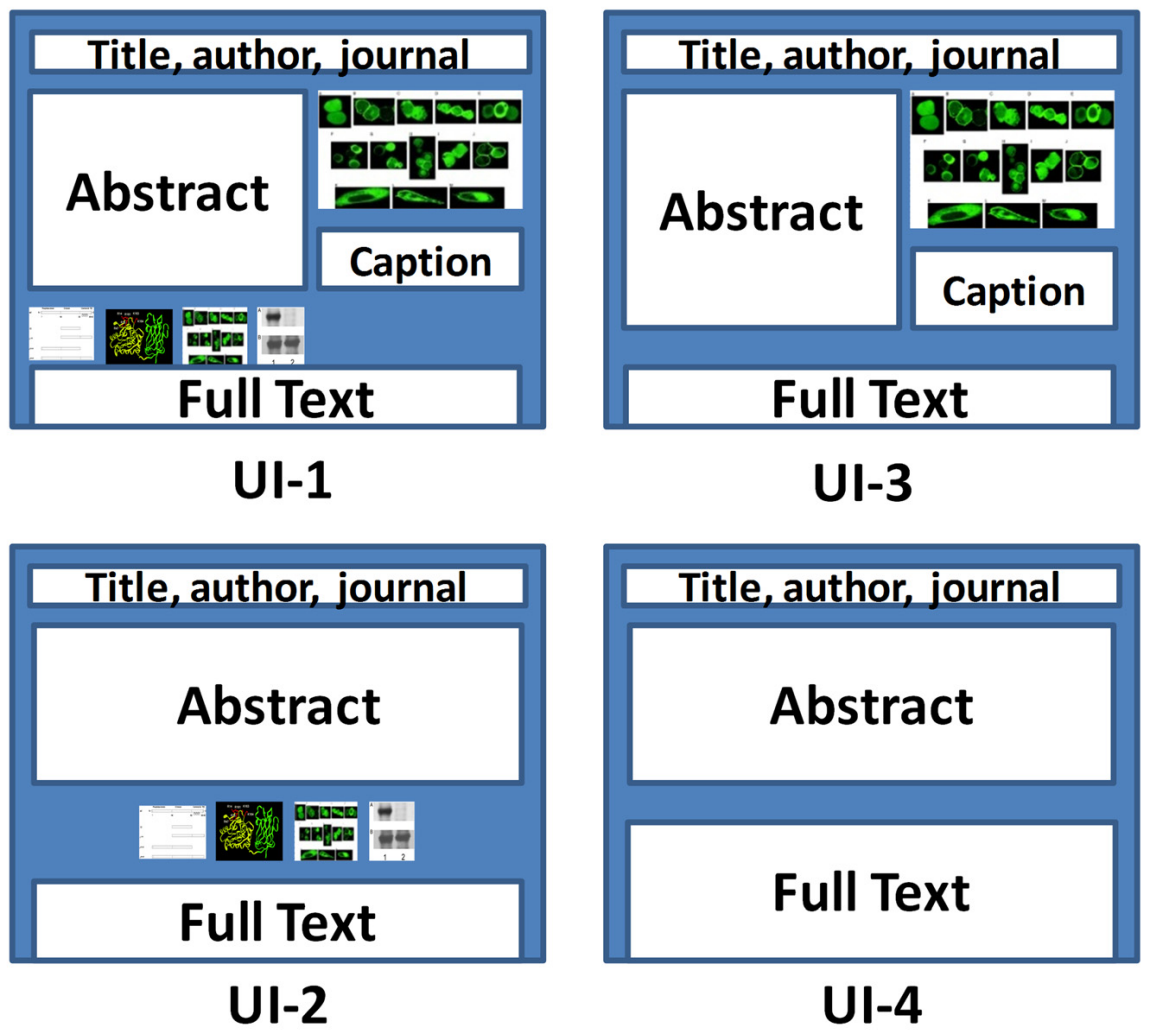
Figure-ranking user interfaces evaluated. UI-1 shows the most important figure enlarged and thumbnails of other figures. UI-2 shows figure thumbnails. UI-3 shows the most important figure only, enlarged. UI-4 shows the full-text article. The full-text article can be accessed from all four UIs.

We speculate that figure ranking can be useful for many text mining tasks, including information retrieval, extraction, and visualization. For example, figure ranking may be incorporated into multi-weighted field information retrieval models (e.g., BM25 [26]), and information extraction (e.g., protein-protein interaction) where higher confidence may be assigned to the events supported by the strongest evidence. In this study, we examined one utility in visualization. Specifically, we hypothesize that bioscience researchers prefer a user interface that highlights the most important figure. As shown in Figure 7, in UI-1 an article is represented by its title, author, journal, and abstract. The user interface also incorporates figure thumbnails. The most important figure is enlarged with its legend shown as well. The user interface also incorporates a link to the full-text article. In order to evaluate the user interface, we also implemented three baseline systems, as shown in Figure 7 UI-2, -3, -4. The first baseline system (UI-2) is similar to UI-1 except that the most important figure is not enlarged and its legend is not shown. The second baseline user interface (UI-3) is similar to UI-1 except that the figure thumbnails have been removed. The third baseline system shows the original full-text article, without the figure thumbnails or the enlarged figure and its legend. A user can access to full-text from any of the four user interfaces.

We randomly chose a subset of 121 articles from the total 252 annotated articles(gold standard collection mentioned earlier), created these four versions of the user interface for each of them, and then asked the authors to rank the user interfaces from most favorite to least favorite. Fifty-eight authors (46%) participated in the evaluation. We excluded three authors who selected all four user interfaces to be their most favorite. The evaluation results of the remaining 55 authors are shown in Table 3.

**Table 3:** Statistical results of user interface(UI) evaluation from 55 participants.

Preference	UI-1	UI-2	UI-3	UI-4
Most favorite	37 (67%)	12 (22%)	5 (9%)	1 (2%)
Second favorite	14 (25%)	22 (40%)	17 (31%)	2 (4%)
Third favorite	3 (5%)	19 (35%)	30 (55%)	3 (5%)
Least favorite	1 (2%)	2 (4%)	3 (5%)	49 (89%)

UI-1: Important figure + thumbnails, UI-2: Figure thumbnails only, UI-3: Important figure only, UI-4: Full text.

As shown in Table 3, the majority of bioscience authors (67%) preferred UI-1, which displays both the most important figure and the figure thumbnails, and 92% (first two rows in first column) of authors preferred UI-1 as the top two choices. The second most popular user interface (rated most favorite by 22%) was the one that incorporates all figure thumbnails (UI-2), while the full-text format (UI-4) was the least popular (rated most favorite by 2% and least favorite by 89%).

We also analyzed the second-favorite user interface choice given the choice of most-favorite user interface. The results, given in Table 4, show the same conclusion: authors preferred the user interface with the enlarged most important figure and other figure thumbnails over just the figure thumbnails or just the most important figure, and the full-text presentation was still the least favorite.

**Table 4:** Probability of user interface being second favorite(the last four rows) given the most-favorite user interface(the first row).

	UI-1(37)	UI-2(12)	UI-3(5)	UI-4(1)
UI-1	-	10 (**83%**)	4 (**80%**)	0 (0%)
UI-2	21 (**57%**)	-	1 (20%)	0 (0%)
UI-3	14 (38%)	2 (17%)	-	1 (**100%**)
UI-4	2 (6%)	0 (0%)	0 (0%)	-

UI-1: Important figure + thumbnails, UI-2: Figure thumbnails only, UI-3: Important figure only, UI-4: Full text.

### Evaluation of UI Enabled by NLP

No NLP systems are perfect. However, a non-perfect NLP system may still be useful. In order to evaluate the utility of our NLP system for figure ranking, we performed the second user interface evaluation: we evaluated three figure ranking systems: author annotated reference, our automatic NLP system(combined system shown in Figure 3), and a random system. We used the remaining 131 articles(excluding 121 articles used in the above section from gold standard collection of 252 articles) for this evaluation. We sent to authors three user interfaces (UIs) respectively based on the figure rankings from aforementioned three systems. All three systems were implemented as the best UI (UI-1 as in Figure 7).

We asked authors to choose preference relationship (better than, as good as, worse than) among three UIs by the three systems. Note that if two of the three systems have the same most important figure, we only sent two UIs instead to authors for evaluation. Currently we obtained responses from 52 authors and the results are shown in Table 5.

**Table 5:** Evaluation of figure ranking by being integrated into novel user interface(UI-1 in Figure 7).

Our System(S) v/s Author Annotated(A)			
Prefer S	Prefer A	Equal	p value
11	19	4	0.144

Our System(S) v/s Random(R)			
Prefer S	Prefer R	Equal	p value
25	12	4	0.033

Author Annotated(A) v/s Random(R)			
Prefer A	Prefer R	Equal	p value
23	8	5	0.007

P values are shown based on Chi-square significance test.

We used chi-square statistics to measure whether the differences are statistically significant. The results show that authors preferred the NLP system than the random one (25 vs. 12), the difference was statistically significant (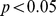). Although authors preferred the author annotated reference than the NLP system (19 vs 11), the difference was not statistically significant. Our results show that author annotation achieved dominant preference than the random system (23 vs. 8) and the difference was statistically significant (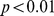).

### A Robust User Interface Design

**Figure 8:**
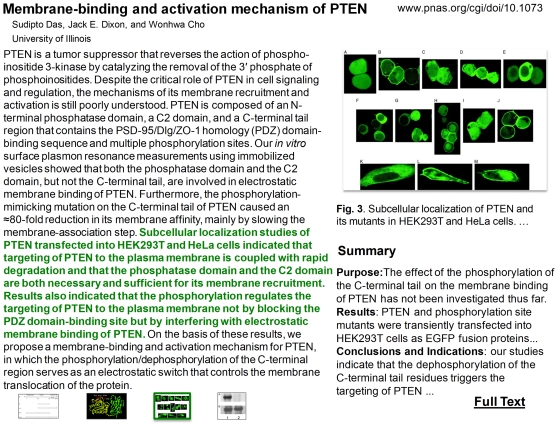
A novel user interface allowing efficient figure access. The user interface shows title, author, reference, abstract, and thumbnails of all figures. When the figure is shown first, the most important figure (“Fig. 3” in this example) is enlarged and highlighted, along with its figure legend and text summary. The corresponding sentences in the abstract are also highlighted. When a user moves the mouse over any figure thumbnail, or any sentence, the corresponding figure is dynamically enlarged, replacing the previous one, and its figure legend and summary are dynamically generated. A link to the full-text article is at the bottom-right corner.

Although our evaluation results(in Table 5) have demonstrated that our NLP figure-ranking system statistically outperformed a random system, and performed close to human annotations, our NLP systems, like any NLP systems, will make errors. In order to cope with a non-perfect NLP system, and to reduce error-consequences, we designed a novel and robust user interface (Figure 8) allowing bioscience researchers to view any enlarged figure easily. The user interface is shown first with the most important figure enlarged. When a user moves the mouse to any of the figure thumbnails, the corresponding figure is dynamically enlarged. In addition, we propose incorporating into the interface the functions of associating figures with sentences in abstract and figure summarization (See Figure 8), as both have been shown to be favored by bioscience researchers [1], [13]. When a user moves the mouse over sentences in the abstract, the corresponding figure is enlarged and its legend and text summary are shown. We speculate that this new user interface will be welcomed by bioscientists, although the UI needs to be formally evaluated.

## Discussion

### Experimental Findings

We have introduced a novel concept of figure ranking and accordingly found that a majority of authors (84.6%) were able to rank their figures in their publications. The results therefore empirically validate that figures appearing in a full-text bioscience article can be ranked by their bio-importance or their contribution to bio-discoveries.

We evaluated our centrality-based unsupervised methods for automatic figure ranking. The methods loosely resemble the approach of the graph-based lexical centrality single document-summarization [17]. Intuitively, the summarization methods apply to our figure ranking because a figure that is more frequently discussed and more widely connected than another figure should be ranked higher. Our results agree with our intuition and show that our methods performed reasonably well, with the best weighted error rate(MWER-RK) to be 0.2. Our application-driven evaluation results demonstrate the effectiveness and feasibility of our NLP system: the best NLP system was significantly preferred by bioscientists to a system that randomly assigned the most important figure, and the best NLP system did not differ statistically from the author annotation.

To obtain annotated data for the evaluation, we worked with over 100 bioscience authors, asking them to rank figures in their publication and evaluate different user interfaces based on figure ranking. Author annotation has been successfully reported previously (KEGG, www.genome.ad.jp/kegg). We found that there is a reverse correlation between an author annotation and the “age” of an article as shown in Figure 1: the older an article, the less likely that the author of the article will respond to our email. The response rate for new publication or the publication within the first year was 27% and dropped to 0% when published articles were seven years old or older. Note that the response rate for new publication was close to 34.7% in our previous study [1]. We did not expect a high response rate as we have requested corresponding authors (who were usually the senior experts with high workload)to perform annotation voluntarily.

The reverse correlation results in author response are not surprising as the biology field is highly evolving and dynamic, and our experience suggest that the best timing for author annotation may be when they are submitting their manuscript for publication. One limitation of author annotation is lack of quality control and inter-annotator agreement. We plan in the future to explore two types of re-annotations on the figure ranking: first, we will ask a co-author of a paper to re-annotate the paper; secondly, we may ask biologists who are not the author of the paper for the re-annotation.

We explored three groups of approaches to model the centrality of figures, including similarity based centrality, frequency based centrality and their combination. Our results show that both centrality modeling approaches achieved overall comparable performance, illustrating different advantages in terms of different metrics. For example, the best similarity based system(*FIGtext*–*ATCabstract*) performs better on MWER-RK(0.223 vs. 0.228) and ER-HR(0.616 vs. 0.649), but worse on MWER(0.322 vs. 0.319) and WER-HR(0.266 vs. 0.249) than the best frequency based system(*WFreqRDAbs*) as presented in Table 1 and 2. It suggests that compared with the frequency based system, the similarity based system produces more error pairs(worse MWER) but tends to correctly rank figure pairs when more important figures are involved(better MWER-RK), and performs better in recognizing the most important figure(better ER-HR) but tends to wrongly assign the much less important figure as the highest rank(worse WER-HR).

Our further analysis of Figure 2 shows that *FIGtext*–*ATCabstract* and *WFreqRDAbs* are exhibiting different patterns in terms of the number of articles at different error rates. We also examined the standard deviation of their performance on different articles, showing that *WFreqRDAbs* has a smaller deviation of 0.223, 0.242, 0.201 and 0.247 on MER, MWER, MWER-RK and WER-HR, respectively, compared to 0.262, 0.266, 0.214 and 0.265, respectively, for *FIGtext*–*ATCabstract*. This difference might be due to the variance of writing styles among articles having a greater effect on the similarity calculation. Motivated by those observations, we combined the two systems and results show that the linear combination can further boost the ranking performance, outperforming all the other systems with a MWER-RK of 0.2 and a WER-HR of 0.232. This implies that frequency and similarity features can compromise their performance behavior difference and interact with each other in a beneficial way to produce better prediction. Note that linear combination is a simple way of incorporating different features. Employing a sound machine learning model to discover the best way of combining different features would be expected to yield much improved performance.

For the similarity based approach, we evaluated different ways of representing both figures and articles. We employed associated contextual information of figures(FIGtext), which is shown to be more helpful than using the figure's legend(*FIGlegend*) as shown in Table 1. This suggests that a figure's associated contextual information contains richer information that is useful for determining the semantic salience among figures.

Our results show that representing articles using abstract(*FIGlegend*–*ATCabstract* and *FIGtext*–*ATCabstract*) performed better, achieving MWER-RKs of 0.265 and 0.223, respectively. This was a performance gain of 6.4% and 12.6%, compared with the corresponding title based systems(0.283 and 0.255 for *FIGlegend*–*ATCtitle* and *FIGtext*–*ATCtitle*, respectively), and a performance gain of 5.4% and 3.9%, compared with full text-based systems(0.28 and 0.232 for *FIGlegend*–*ATCtext* and *FIGtext*–*ATCtext*, respectively). This may be because the abstract incorporates more topic essential information than the title while avoiding other noise from the full text.

However, in terms of recognizing the most important figure only, using the title information achieved the best ER-HR of 0.594 and WER-HR of 0.246(6th row in Table 1), showing that the article title may contain information that is more beneficial for determining the most important figure.

For the frequency based approach, we investigated leveraging frequency information in various ways as in Table 2. We found that the frequency in the full text(*FreqFullText*) was very important for figure ranking. This is not surprising as frequency features are essentially the heart of both single and multi-document summarization [17], [27].

We refined the figure frequency information by limiting it to only results and discussion(R&D) sections and integrating topic analysis. The rationale is that most bioscience articles are organized by the IMaRD format (Introduction, Method, Result, and Discussion) [14] and that the R&D sections are the likely sections in a bioscience paper to discuss novel and important research findings. The experimental results show that only using R&D frequency information(*FreqRD*) slightly degraded the performance(not statistically significant), but when further integrated with the topic analysis, interpolated frequency information weighted by topical salience score *WFreqRDAbs* yielded the best MWER-RK of 0.228, which was 9.5% better than 0.252 obtained by directly using the frequency in results and discussion sections(*FreqRD*), and 8.4% better than 0.249 obtained by using the full text frequency. This validates our hypothesis that assigning more weights to the frequency information in the more topic-indicative paragraph will boost the ranking performance, which also shows a promising future direction to apply topic modeling or latent semantic analysis in the figure ranking task. We also observed that approaches based on the most topic-related referring paragraph(*WFreqRDParaTitle* and *WFreqRDParaAbs*) didn't perform as well as the interpolated ones(*WFreqRDTitle* and *WFreqRDAbs*), which is probably due to the lack of necessary contextual information.

We introduced two new metrics(MWER-RK and WER-HR) for evaluating our different automatic figure ranking systems. We can see from Figure 2 that MWER-RK is showing better performance, which suggests that a significant portion of the wrong predictions includes figures at relatively lower ranks—the less important figures and it also proves the necessity of using MWER-RK for more reasonable evaluation. Similarly, MWER shows advantages over MER indicating systems tend to make errors when figures are closer in reference ranking than when they are far away and in those cases MER will provide a biased evaluation. As to recognizing the most important figure, WER-HR provides a more reasonable way to assess the performance of ranking systems by considering the distance between the most important figure and the system prediction for each article.

We evaluated one application of our figure ranking system, which is a novel user interface to show the enlarged, most important figure when scientists browse an article. Our evaluation results show that over 67% scientists prefer a user-interface incorporating the most important figure and other figure thumbnails(UI-1). We found that less preference were given on UI-3 including the most important figure only than UI-2 including all the thumbnails only, which we speculate is due to the fact that figure thumbnails cover more information than the most important figure alone. The fact that over 92% authors prefer UI-1 as top two choices among 4 UIs strongly support our hypothesis of integrating ranked figure into a novel user interface.

On the other hand, our error analyses have also shown significant challenges in figure ranking task, and therefore it is unlikely to develop a perfect automatic figure-ranking system although we may need to explore new methods (see Conclusion and Future Work, below) for further performance improvement. Before that, instead of using reference ranking in the first UI evaluation, we conducted another UI evaluation using automatic figure ranking, which shows that our current ranking system significantly outperformed (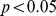; Chi-square test) the baseline system in which the most important figure is randomly assigned, and most importantly, there is no statistically significant difference (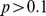; Chi-square test) between our system and the gold standard assigned by domain experts. It suggests that our non-perfect automatic figure ranking system can still be of significant utility to bioscience researchers when being integrated into our novel user interface, although more extensive evaluation is still needed for further validation. This also further proves the effectiveness of both our automatic figure ranking and proposed new UI as well as the feasibility and robustness of integrating them to facilitate information browsing in biological domain.

Several challenges remain in our current system. One challenge is preprocessing articles with different structures and writing styles, which might create some noise when extracting structural information for both figures and articles. The wrongly processed documents will introduce errors. Another challenge is that it is very challenging to improve the performance of recognizing the most important figure according to our experimental results. The best ER-HR of 0.594 is still rather low, although much better than the baseline of 0.831 when randomly selecting one out of the average 5.9 figures per article. In addition to the causes we discussed in the error analysis section, we found that our preprocessing failed to accurately associate some figures to texts which could explain a portion of the errors. Finally, it is very difficult to develop an optimized model that can work reasonably well for most articles. We found that for the figure ranking task different articles favor different approaches quite differently, and some approach can perform much better or much worse on certain subsets of articles, implying that a customized figure ranking model should be considered to address the challenging diversity of bioscience articles.

### Conclusion, Limitation, and Future Work

We empirically tested a novel concept that figures appearing in a full-text bioscience article can be ranked and explored unsupervised approaches for automatically ranking figures, with the best yielding a MWER-RK error rate of 0.2 and a WER-HR error rate of 0.232 in the combined system. One limitation of our work is that figures were ranked by the author of an article. In future work, we will explore methods to allow multiple domain-experts to rank figures and then evaluate inter-rater agreement. Our current algorithms are designed to be generic or domain-independent. Although generic systems have their advantages, the performance may be further improved with domain-specific adaptations. In the future, we may group articles by their sub-domains (e.g., molecular biology, structural biology, and system biology) and explore and evaluate approaches in each sub-domain.

We will further explore NLP approaches, including rich features, other alternative textual similarities, and supervised learning approaches that has been applied on ranking and reranking tasks. For example, machine learning on ranking (also called ordinal regression) and reranking has been applied to many tasks, such as speech recognition [28], [29], information extraction [30], [31], information retrieval [32], [33], question answering [34], syntactic parsing [35]–[37], machine translation [38], [39], and gene prediction [40]. Such learning based method has also been explored extensively in figure ranking tasks including ImageCLEF, the evaluation competition of cross language image retrieval as part of the Cross Language Evaluation Forum (CLEF) [41]. However, we recognize that approaches explored for ranking problems, including cumulative ordinal regression model from statistics perspective [42], perceptron learning [43], Gaussian processes model [44], support vector machines-based regression [45]–[47], and classification approaches [48]–[50], were mostly developed on ranking objects in the scope of whole training and testing data. This is not the case in our figure ranking task, where figures are ranked within each article. Therefore, we will explore supervised methods on reranking which reranks N-best candidates for each object of interest, similar to ranking figures in each article, such as boost loss model and log_likelihood loss model [35], perceptron learning [51], and support vector machines with tree kernels [52].

We evaluated the novel user interface, which shows that 92% of the bioscience researchers preferred, as top two choices among the 4 UIs, the interface (UI-1) in which figure thumbnails are shown and the most important figure is enlarged. Our user evaluation results also show that our non-perfect figure ranking system is highly preferred by bioscience researchers. Future work we will apply our user interface to the PubMed Central open access articles and evaluate its utility. Furthermore, we will explore approaches to incorporate figure ranking to improve information retrieval in the Genomics domain.
